# Oropharyngeal swallowing hydrodynamics of thin and mildly thick liquids in an anatomically accurate throat-epiglottis model

**DOI:** 10.1038/s41598-024-60422-x

**Published:** 2024-05-25

**Authors:** Amr Seifelnasr, Peng Ding, Xiuhua Si, Andres Biondi, Jinxiang Xi

**Affiliations:** 1 Department of Biomedical Engineering, University of Massachusetts, 1 University Ave., Falmouth Hall 302I, Lowell, MA 01854 USA; 2 Department of Otolaryngology-Head and Neck Surgery, Cleveland Clinic Lerner College of Medicine, 9501 Euclid Ave, Cleveland, OH 44195 USA; 3 Department of Mechanical Engineering, California Baptist University, 8432 Magnolia Ave, Riverside, CA 92504 USA; 4 Department of Electrical and Computer Engineering, University of Massachusetts, 1 University Ave., Lowell, MA 01854 USA

**Keywords:** Oropharyngeal swallowing, Epiglottis, Fluid bolus hydrodynamics, Aspiration, Biological techniques, Biophysics, Biotechnology, Physiology, Anatomy, Diseases, Health care, Medical research, Signs and symptoms, Engineering

## Abstract

Understanding the mechanisms underlying dysphagia is crucial in devising effective, etiology-centered interventions. However, current clinical assessment and treatment of dysphagia are still more symptom-focused due to our limited understanding of the sophisticated symptom-etiology associations causing swallowing disorders. This study aimed to elucidate the mechanisms giving rise to penetration flows into the laryngeal vestibule that results in aspirations with varying symptoms. Methods: Anatomically accurate, transparent throat models were prepared with a 45° down flapped epiglottis to simulate the instant of laryngeal closure during swallowing. Fluid bolus dynamics were visualized with fluorescent dye from lateral, rear, front, and endoscopic directions to capture key hydrodynamic features leading to aspiration. Three influencing factors, fluid consistency, liquid dispensing site, and dispensing speed, were systemically evaluated on their roles in liquid aspirations. Results: Three aspiration mechanisms were identified, with liquid bolus entering the airway through (a) the interarytenoid notch (notch overflow), (b) cuneiform tubercle recesses (recess overflow), and (c) off-edge flow underneath the epiglottis (off-edge capillary flow). Of the three factors considered, liquid viscosity has the most significant impact on aspiration rate, followed by the liquid dispensing site and the dispensing speed. Water had one order of magnitude higher aspiration risks than 1% w/v methyl cellulose solution, a mildly thick liquid. Anterior dispensing had higher chances for aspiration than posterior oropharyngeal dispensing for both liquids and dispensing speeds considered. The effects of dispending speed varied. A lower speed increased aspiration for anterior-dispensed liquids due to increased off-edge capillary flows, while it significantly reduced aspiration for posterior-dispensed liquids due to reduced notch overflows. Visualizing swallowing hydrodynamics from multiple orientations facilitates detailed site-specific inspections of aspiration mechanisms.

## Introduction

Swallowing is a complicated and coordinated process involving many muscles and nerves that work together correctly to ensure safe and efficient food/drink transport from the mouth to the stomach^1^. Dysphagia denotes swallowing difficulties arising from issues related to the throat, esophagus, or even the brain and nerves governing the swallowing process^2^. It ranges from mild discomfort to a complete inability to swallow. Oropharyngeal dysphagia is an underlying pathology occurring during the oropharyngeal phase and can significantly affect an individual's ability to speak, swallow, or breathe^3^. Approximately 5% of the adult population worldwide suffers from dysphagia^4,5^. Patients with dysphagia are three times more likely to develop pneumonia because dysphagia can result in aspiration, i.e., food or drink entering the larynx and lower respiratory tract^6,7^. Aspiration pneumonia accounts for 67% of hospitalizations due to pneumonia and has been a leading cause of death among residents of nursing homes^8^. The aspiration pneumonia-associated death rate drastically increased with age, from 1% for 40 years old and younger to 44% for 85 + years old^9^. Aspiration pneumonia is responsible for more than 61,829 fatalities annually in the United States^9^.

Common causes of oropharyngeal dysphagia include acute disorders such as stroke and head trauma, neurodegenerative diseases (e.g., Alzheimer's, Parkinson's, and multiple sclerosis), neuromuscular disorders such as polymyositis, and local/structural lesions such as surgical pharyngeal resection^10–12^. Current treatments and management of dysphagia, with increasing severities, include swallowing rehabilitation, dietary alterations, feeding tubes, and surgeries. The Shaker exercise strengthens the traction forces of suprahyoid muscles through a regimen of head raises from supine^13^. Dietary alterations modify food preparation and omit those that present a challenge for swallowing^14,15^. Surgical approaches include laryngeal suspension, upper esophageal sphincter myotomy, unilateral vocal cord medialization, etc. Laryngectomy, which is considered the final resort procedure, also has the risk of losing the ability to speak^16^. It is noted that these treatments more often focus on clinical impairments or symptoms rather than the etiology of disordered swallowing^17^.

Many factors make it challenging to study swallowing dynamics and dysphagia and find effective interventions. First, swallowing is highly dynamic. A food or fluid bolus passes through the pharynx in less than 1 s, accompanied by pharyngeal peristalsis at 40 cm/s^18^. Second, swallowing involves precisely sequenced and coordinated muscle movements. It is difficult to capture the motions of all structures using 2D videofluoroscopy^19^. On the other hand, CT (4D or 3D) or dynamic MRI does not have a sufficient acquisition rate to fully capture the rapid motions of participating structures. For the same reason, we have not yet found any high-resolution CT images with a down-flapped epiglottis representing swallowing, but only images with an upright epiglottis representing respiration. Its kinematics can only be viewed from a 2-D videofluoroscopy or a 3-D endoscopy with a top view, neither of which can completely depict the complex motions of the epiglottis, pharynx, and larynx. The third factor is the multi-faceted symptoms and etiologies of dysphagia, where seemingly similar symptoms can result from different etiologies, and the same etiology can lead to distinct symptoms. This sophisticated symptom-etiology association makes it difficult to accurately diagnose underlying neuromuscular disorders and devise an individualized plan of intervention. The fourth factor hindering dysphagia research is its multidisciplinary nature, which intertwines anatomy, physiology, engineering, and neurological sciences. What makes it difficult to study from an engineering perspective includes the intricate geometry, sequentially coordinated kinematics, two-way coupled fluid–structure interactions, non-Newtonian fluids with a wide range of viscosities, hyperplastic materials, and multi-body dynamics. Computationally, these factors still remain challenging to model and resource-intensive to simulate^20–25^. Experimentally, it is difficult to replicate multi-component, synchronized motions using electro-mechanical (robotic) systems^26–33^.

Despite a wealth of descriptions of swallowing-related anatomy and clinical symptoms, there is less information available on how anatomy causally relates to clinical presentations. Aspiration can result from various etiologies, including structural abnormalities, neurological disorders, or weakened swallowing muscles. The underlying mechanisms can be complex and hierarchical, with similar symptoms arising from varying pathophysiologies or an identical pathophysiology leading to distinct symptoms. It has been advocated that dysphagia interventions should be etiology-centered rather than symptom-focused, as is common in current practices^17^. However, without a clear understanding of the underlying mechanisms for aspiration, interventions will remain empirical and speculative. Given the rapid and intricate nature of swallowing, which is too swift for the naked eye to discern its progressive stages and assess their structural–functional relationships, Fanucci et al.^34^ suggested a sequential breakdown of the swallowing process for detailed analyses. The slow-motion mode, ‘freeze’ images, and rewinding permitted a close examination of the structural kinematics and the hydrodynamics of a food or liquid bolus. Integrating temporal and spatial data, as well as the structure and bolus motions, enables a thorough assessment of the swallowing process. Gardon et al.^35^ identified seven distinct epiglottic patterns in 500 patients with varying etiologies, with each pattern giving rise to varying rates and amounts of aspiration. Note that swallowing fluids involves a slightly different coordination of muscles and timing compared to food boluses, which are chewed solids or semi-solid mixed with saliva.^36^ The aspiration risks are higher with fluids as they move quicker and are less controllable than solid boluses^37^. Thus, it is of great importance to understand the mechanisms underlying these aspiration risks, with the aim of devising pertinent interventions to mitigate such risks.

The objective of this study was to investigate the factors influencing aspiration risks during swallowing in anatomically accurate human throat models. Fluid bolus dynamics passing the epiglottis and leading to dysphagia were visualized from different orientations to identify the underlying aspiration mechanisms. Specific aims included:

1. Develop two throat models (full throat and half throat) with an epiglottis at different angles.
2. Visualize liquid dynamics in the full-throat and half-throat models with a 45° down-tilt epiglottis from different directions (lateral, rear, front, and inside).
3. Systemically assess aspiration risks under the influence of different liquid consistencies (thin *vs*. mildly thick), liquid dispensing sites (anterior oropharynx *vs*. posterior oropharynx), and liquid dispensing speed (fast vs. slow).
4. Identify the underlying mechanisms for aspiration under different dispensing conditions.

## Materials and methods

### Throat-epiglottis models

**Figure 1:**
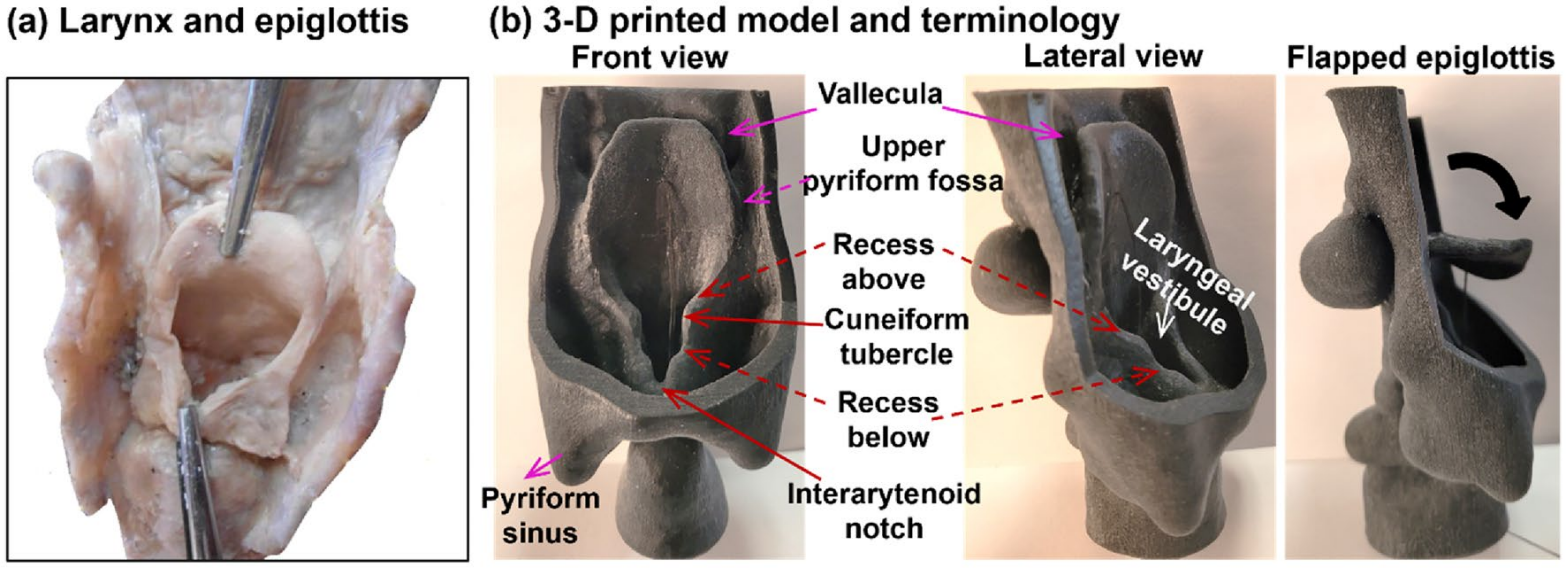
Throat-epiglottis anatomy and model: (**a**) anatomy of the larynx and epiglottis adapted from^40^, and (**b**) an image-based throat-epiglottis model with landmark structures affecting swallowing including the vallecula, upper pyriform fossa (the groove between the lateral pharynx and epiglottis), cuneiform tubercle, recesses above/below the cuneiform tubercle, interarytenoid notch, and pyriform fossa (sinus). The laryngeal vestibule is closed (or partially closed) by the down-flapped epiglottis during swallowing and opens with the epiglottis tilted up.

The throat-epiglottis models used in this study were modified from a respiratory tract model that was originally constructed from high-resolution CT images of an 18-year-old male (weighting 72 kg) by Corley et al. in 2015^38^. The CT images had an isotropic resolution of 623 µm and captured fine respiratory-swallowing anatomical details, including the uvula, epiglottis, laryngeal vestibule, and glottis^38,39^. The usage of the CT-based model to generate throat-epiglottis hollow casts was exempted by the Institutional Review Boards of the University of Massachusetts, Lowell. Figure 1 shows the 3D-printed throat-epiglottis model using black rubber in comparison to an in vivo specimen^40^, as well as associated terminologies needed to explain the aspiration mechanisms. The dorsal pharynx was removed to reveal the epiglottis and neighboring anatomy. It was evident that the current model faithfully retained all essential structures associated with deglutition/swallowing. First, a close resemblance was observed between the cadaver-extracted specimen and the CT-reconstructed model, particularly for the leaf-shaped epiglottis and the laryngeal vestibule beneath it that opened to the glottis. An event of aspiration or penetration was considered to occur if any liquid entered the laryngeal vestibule, regardless of whether it entered through the lowest interarytenoid notch, the recess above or below the cuneiform tubercle, and or aryepiglottic fold (epiglottis edge). The flow channels or reservoirs in this region included (1) the vallecula, which was the space between the epiglottis based and tongue, (2) the two upper pyriform fossae (i.e., the two lateral channels between the epiglottis and the side pharyngeal walls), and (3) the two lower pyriform fossae (or sinuses) that served as the buffer zone for the fluid bolus before draining into the esophagus. During a normal swallowing event, the epiglottis quickly flaps downward, covering the entrance to the laryngeal vestibule, and then returns to the upright position after swallowing. The rightmost panel of Fig. 1b shows the epiglottis being manually flapped downward from the resting, upright epiglottis (third panel in Fig. 1b).

**Figure 2:**
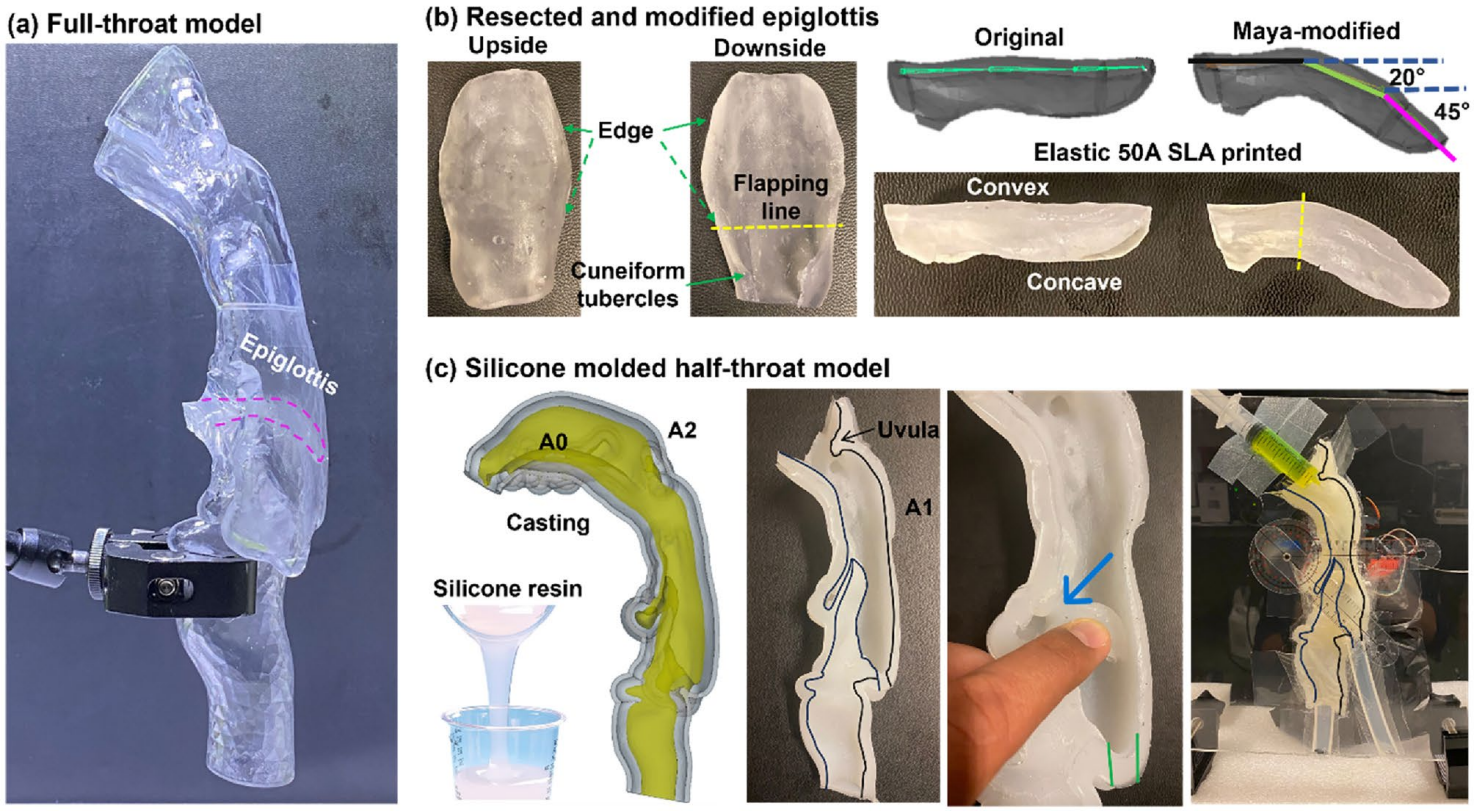
Experimental setup and models: (**a**) full-throat model printed using an elastic 50A SLA material, (**b**) resected and modified epiglottis model tilted downward at 45°, and (**c**) silicone-molded half-throat model.

Two methods were used in this study to simulate the epiglottis's orientations during deglutition. In the first method, the leaf-shaped epiglottis was separated from the throat model as an individual entity from the base of the tongue (Fig. 2b). The pharyngolaryngeal passage (Fig. 2a) was 3D-printed using clear stereolithography (SLA) resin (Formlabs Clear Resin, Somerville, MA), while the resected epiglottis (Fig. 2b) was 3D-printed separately using a transparent flexible material, Elastic 50A Resin (Formlabs, Somerville, MA). The 3D printer was a Form 3BL (Formlabs, Somerville, MA) with a printing resolution of 25 µm. Thus, the surface roughness and quality were highly faithful to the 3D geometry segmented from the CT scanned images. Under careful visual scrutiny, there were no visually observable deviations between the segmented model and the printed one. This was also the case with the silicone molded half-throat model, as the molds were 3D printed using the same high-fidelity printing technique. Before each experiment, these two parts were reconnected, with the epiglottis being inserted through the ventral pharynx and oriented at 45^o^ from the horizontal without touching the dorsal pharynx. Special care was taken to avoid leaks at the interface between the epiglottis and the ventral pharynx. To achieve this, a clear sealant (Shoe Goo, Eugene, Oregon) was used to seal any cracks along the interface, and it was left to fully solidify for 5 h or more. To simulate the downward curved epiglottis, Autodesk Maya (Autodesk, San Francisco, CA) was used to bend the epiglottis from the horizontal to 45°, with the middle section bending 20° from the horizontal, as shown in Fig. 2b. Note that the maximal tilt angle of the epiglottis differs between health and dysphagia; even for healthy participants, the maximal tilt angle can vary from 15° to 80^o^^35,41^. A down-folded epiglottis of approximately 45° has been frequently observed during swallowing in videofluoroscopy images^42–45^. In particular, Seo et al. reported a maximal epiglottis tilt angle of 134° ± 13° from the y axis from a cohort of 35 post-stroke non-aspirating patients^41^. This angle is equivalent to 44° ± 13° down-tilt from the horizontal and examining a 45° down-tilt epiglottis is promising to shed light on the most frequent swallowing scenarios in stroke patients^41^.

In the second method, a mold casting technique was used to prepare the half-throat model (right side only), as shown in Fig. 2c. First, the original mouth-throat model (A0, yellow) was expanded by 3 mm in thickness to create model A1, and A1 was expanded by an additional 3 mm to create model A2 (grey color). Liquid silicone (LET’S RESIN, HongKong) was then poured into the space between A0 and A2 and allowed to sit for twelve hours before solidifying, resulting in a flexible cast A1 (white color). The half-epiglottis can be bent to a wide range of angles, as illustrated in the third panel in Fig. 2c. The half-throat geometry was then fixed to plexiglass, through which both the anatomical structures and fluorescent fluids could be clearly visualized.

### Fluid property characterization

Water and 1% w/v methyl cellulose (MC) solution were chosen as test liquids in this study. This selection was based on IDDSI (International Dysphagia Diet Standardization Initiative) testing of MC solutions at varying concentrations, as listed in Table 1. The 1% w/v MC solution was categorized as mildly thick (Level 2), while water was classified as thin or Level 0. Consequently, distinct hydrodynamic behaviors in the throat-epiglottis models and distinct responses to varying swallowing conditions were expected between these two fluids^46^. We also tested 2% w/v MC solution but observed no aspiration with anterior/posterior and slow/fast dispensing. Considering that the major aim of this study was to identify aspiration mechanism, test results using 2% w/v MC solution were thus excluded.

**Table 1:** International Dysphagia Diet Standardization Initiative (IDDSI) liquid thickness category testing according to the volume of liquid remaining in 10 mL funnel after 10 s. MC: methylcellulose.

Formulation	Remaining volume after 10 s (mL)	IDDSI Category
Water	0	0
0.7% w/v MC	3.8	Level 1
1.0% w/v MC	7	Level 2
1.1% w/v MC	8.2	Level 3
1.2% w/v MC	9.1	Level 3
2.0% w/v MC	9.95	Level 3

The liquid properties and their interactions with the model materials were evaluated in terms of viscosity, surface tension, and wall contact angle. A digital rotational viscometer (Cgoldenwall, NDJ-5S) was used to measure liquid viscosity. Measurements were made at different rotation speeds using appropriate spindles to account for the shear-thinning effect of the MC solution^47^. Surface tension was quantified using a surface tensiometer apparatus (American 3B Scientific, Tucker, Georgia, U.S.A.), with each measurement repeated at least three times.

**Figure 3:**
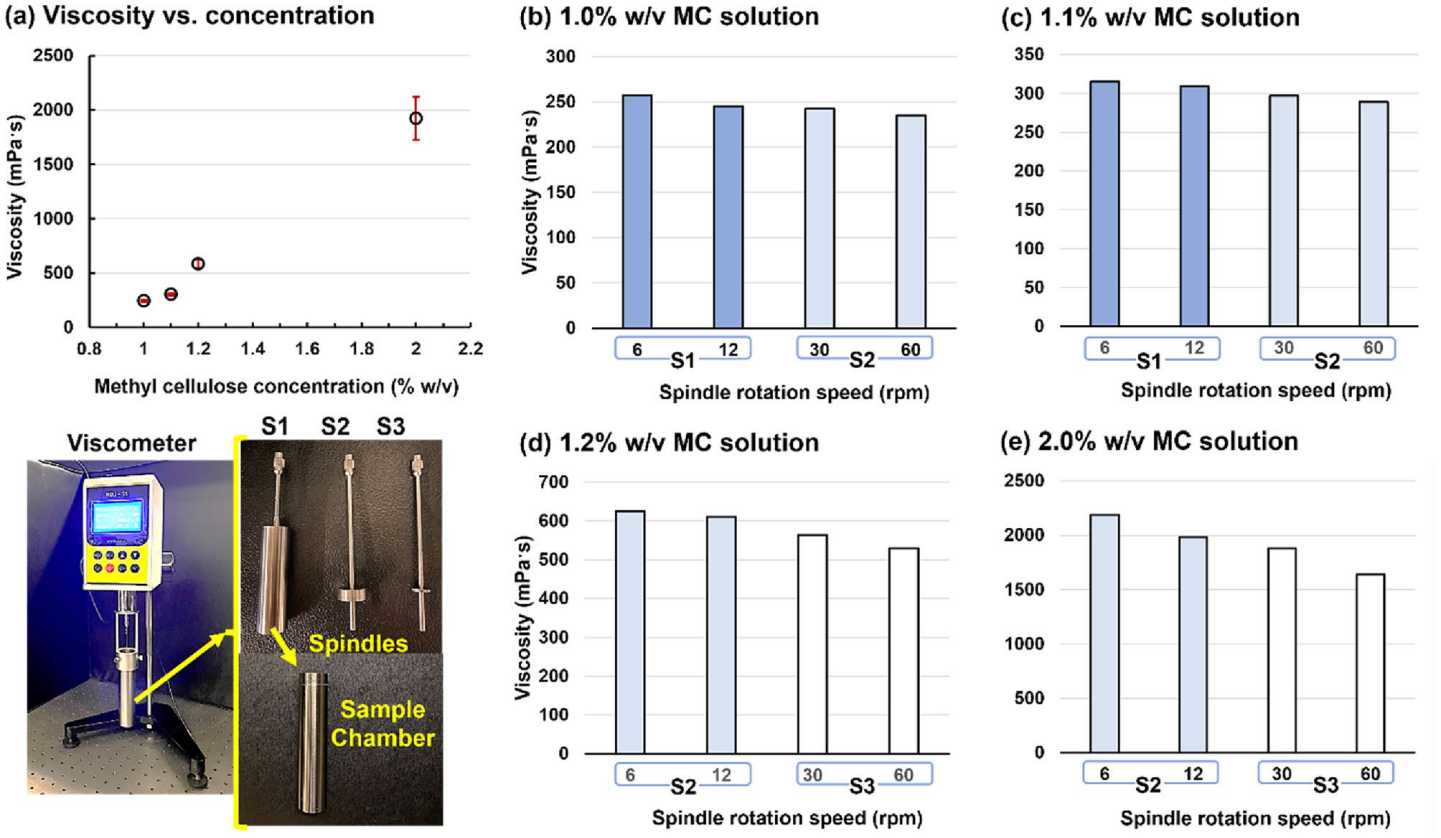
Formulation viscosities: (**a**) viscosity vs. concentration and shear thinning effects for methylcellulose (MC) solutions with a concentration of (**b**) 1.0% w/v, (**c**) 1.1% w/v, (**d**) 1.2% w/v, and (**e**) 2.0% w/v.

Contact angles of liquids on different materials were measured using a custom contact angle gauging apparatus (Fig. 3a). Liquid drops were released from a Hamilton 81,242 500 µL threaded plunger syringe (Hamilton, Reno, Nevada, USA) equipped with a Metcal 922,050-TE 0.5 in, 22-gauge stainless steel blunt tip needle (Metcal, Cypress, California, USA). The drops were dispensed onto a flat sample material placed on top of a horizontally leveled platform. Drop volume was precisely controlled via the threaded dispenser of the plunger syringe. The platform featured a mechanically adjustable height, which allowed for accurately maintaining a fixed distance between the needle tip and the sample surface.

### Study design and experimental procedures

It is hypothesized that using an anatomically accurate throat geometry, even with a static epiglottis, could shed valuable light on the fundamental mechanisms that cause aspiration or dysphagia. It is also hypothesized that the knowledge learned from these simplified models could be extrapolated to live swallowing process or refined when the dynamic components are included in the throat model in the future. We aimed to identify fundamental fluid dynamics features leading to aspiration and understand the mechanisms underlying these risk factors. To evaluate the effects of liquid consistency on aspiration risk, water (thin, Level 0) and a 1% w/v MC solution (mildly thick, Level 2) were tested. To evaluate the tongue retraction effects, the liquid was dispensed to two sites: the posterior oropharynx representing a normal tongue retraction and the anterior oropharynx representing a weaker tongue retraction^48–51^. Dispensing to the side oropharynx was also considered for comparison purposes. To evaluate the swallowing effort, the liquid was dispensed at two speeds (i.e., fast and slow, or dispensing 5 mL liquid within 1 s and 3 s)^52–54^. Each test was repeated at least three times to compute the aspiration mean and standard deviation. For all these tests, the epiglottis was positioned at a 45° down-tilt from the horizontal direction.

To obtain a more comprehensive view of the fluid motions around the epiglottis, both the full-throat model and half-throat model were tested, with the former providing an unprecedented 3D fluid pattern while the latter provided a mid-sagittal view similar to videofluoroscopy. Leveraging the transparency of the full-throat model, rear and front views were also recorded to further examine the flow motions near the laryngeal vestibule and epiglottis.

Water-soluble fluorescent green dye (GLO Effex, Murrieta, CA, USA) was mixed with liquid solutions to visualize liquid dynamics. Videos were recorded in a low-light setting using a UV purple LED light with a wavelength of 385–395 nm. To quantity aspiration, liquids were collected under the trachea and esophagus and weighed using an electronic scale (120 g/0.0001 g, Bonvoisin, A&D Medical, San Jose, CA). Sufficient time after dispensing was allowed for the fluid to drain out of the model, specifically for any liquid passing through the trachea (i.e., aspiration). The waiting time was longer for the 1% MC solution than for water. At least 3 to 4 min were allowed after dispensing the MC solution, whereas the waiting time for water was around 1 min. Before removing any tray for weighing, a visual inspection was performed to ensure that there were no more liquid drops or film moving along the inner walls of the trachea or esophagus towards the tray.

### Statistical analyses

Each assessment was conducted a minimum of three times. Data analysis was carried out using Minitab (State College, PA, USA). We utilized a one-way analysis of variance (ANOVA) to evaluate variations in the results. The aspiration rate was presented as the mean ± standard deviation. To compare the impacts of influencing factors such as liquid type, dispensing site, and dispensing speed, a two-tailed test was used to calculate the p-value.

## Results

### Liquid viscosity and liquid-wall contact angle

#### Methyl cellulose solution viscosities

Figure 3 shows the measured viscosity of Methyl Cellulose (MC) solutions at varying concentrations (1–2% w/v). The mean viscosity increased quickly with the MC concentration, from 245 ± 8 at 1%, to 1922 ± 197 mPaˑs at 2%. To consider the shear-thinning effect, the MC solutions were measured at different rotation rates with corresponding spindles (Fig. 3b–e). For each solution, the viscosity decreases with increasing shear rate. However, the slope of their profiles differs significantly, which is approximately − 0.41, − 0.48, − 1.76, and − 10.1 mPaˑs/rpm for 1%, 1.1%, 1.2%, and 2% w/v MC solutions, respectively (Fig. 3b–e). According to the Pureed texture categorization^55–58^, the 1% w/v MC solution are still thin liquid but closely approaching ‘nectar” consistency liquid (i.e., 300–1500 mPaˑs). The 2% w/v MC solution belongs to “honey” consistency thickened liquid that has a range of 1500–3000 mPaˑs.

The surface tension was measured to be 72.11 ± 0.37 mN/m for water and 79.81 ± 0.86 m N/m for 1% w/v MC solution. Different liquid behaviors are expected, particularly in confined spaces like the vallecula and esophagus opening, where surface tension influences liquid bank formation or breakup. A higher surface tension may cause liquid bridging over the esophagus opening and prevent the liquid from entering the esophagus, while a lower surface tension may facilitate a smoother entrance.

#### Contact angle of MC solutions on model materials

**Figure 4:**
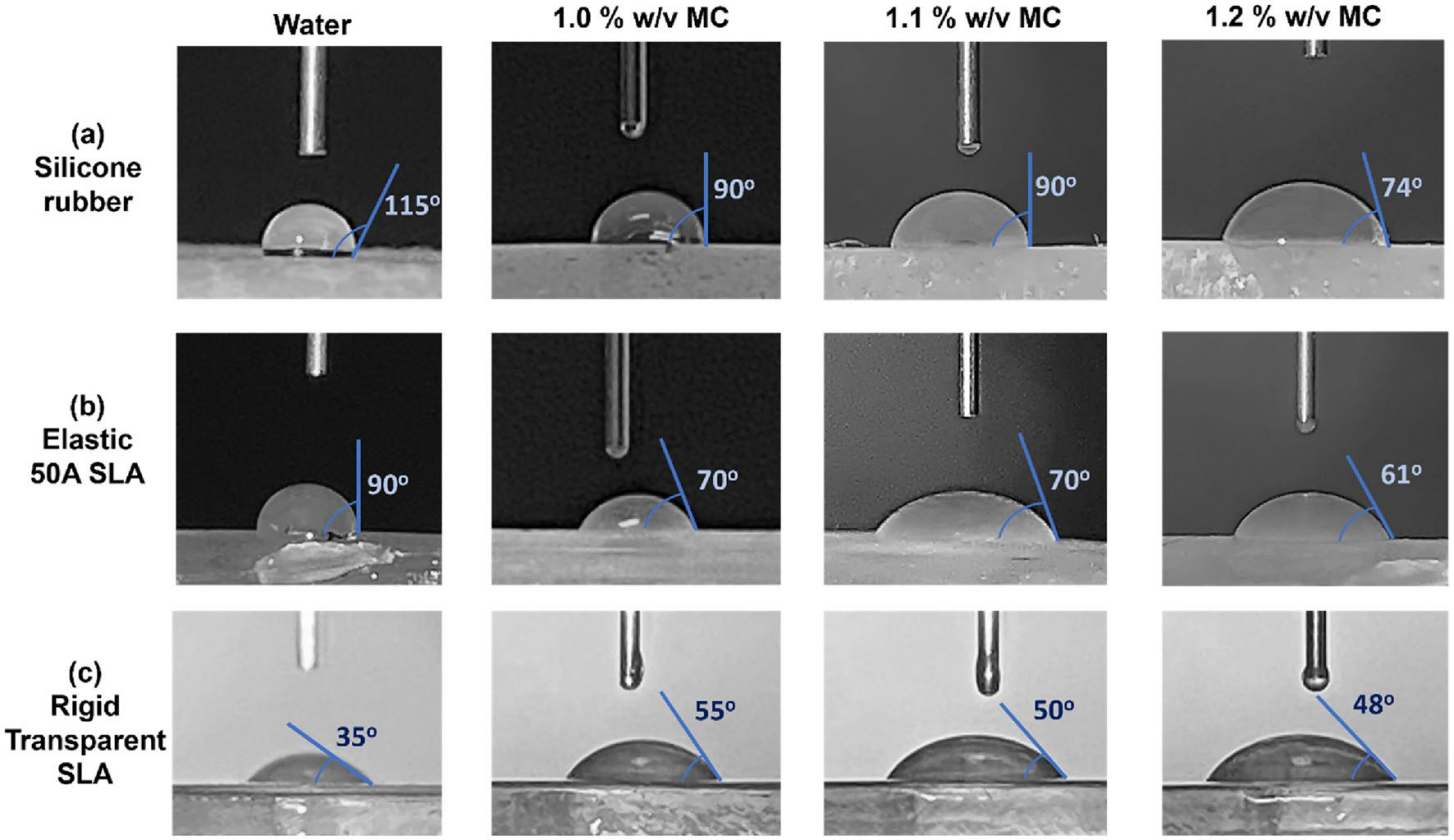
Contact angles for various formulations on: (**a**) silicone rubber, (**b**) elastic 50A SLA, and (**c**) rigid transparent SLA.

Figure 4 shows the contact angles on the three materials for water and MC solutions with three concentrations (1%, 1.1%, and 1.2% w/v). For all liquids considered, the silicone rubber exhibited the highest contact angle, followed by the elastic 50A SLA, while the rigid transparent SLA demonstrated the smallest angle, indicating a superior wettability on the rigid transparent SLA than on the other two materials. Water had a larger contact angle than the MC solutions on the silicone rubber and elastic 50A SLA, but a lower contact angle on the rigid transparent SLA. In particular, the water contact angle decreased drastically from silicone rubber (115°) to elastic 50A SLA (90°), and then to rigid transparent SLA (35°). A similar trend was also found for MC solutions but with a smaller decrease in magnitude. Moreover, the MC solution contact angle decreased slightly with increasing concentration for the three materials considered. The variations in contact angle indicate different fluid behaviors on various test materials, highlighting the need to consider potential differences in fluid behaviors between in vivo tissues and in vitro materials.

### Deglutition tests using 50A SLA full-throat model

Liquid motions and aspirations were systemically studied under four dispensing conditions: fast posterior, slow posterior, fast anterior, and slow anterior. Each condition was repeated at least three times. Negligible aspirations were observed under the slow posterior dispensing condition. With the aim of identifying factors leading to aspiration, only results of the other three conditions are presented in the following sections.

#### Fast dispense of water to the posterior oropharynx

**Figure 5:**
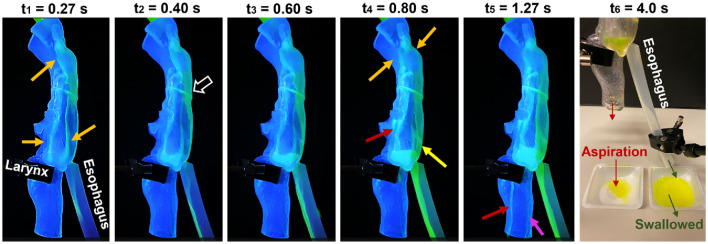
Temporospatial hydrodynamics of water being fast dispensed to the posterior oropharynx. Also shown in the supplemental video recording S1.

Figure 5, as well as the supplemental video S1, shows the hydrodynamics for fast dispensing of water (i.e., 5 mL within 1 s) to the posterior oropharynx. The fast-dispensed water was deflected upon impaction and bifurcated into two streams. The curved oropharyngeal wall was directly above the pharyngeal space, which facilitated the deflected water to reach the tongue base. This small portion of water streamed downward within the upper pyriform fossae (i.e., the groove between the lateral pharynx and epiglottis), entering the lower pyriform fossa (or sinus); an even smaller portion entered the laryngeal vestibule via the recesses above and below the cuneiform tubercle. The latter caused a stream of aspiration, as shown in the middle lateral wall of the trachea (red arrow, fifth panel, Fig. 5). Due to fast dispensing, the water line in the pyriform fossa rose beyond the interarytenoid notch, leading to overflow through the notch, forming a stream along the dorsal trachea (pink arrow, fifth panel, Fig. 5).

On the other hand, the majority of water flowed down along the dorsal pharynx wall and accumulated in the pyriform fossa before entering the esophagus, as illustrated in the first five panels. This process was quick, lasting 1.27 s till the fifth panel. The 45°-down-tilt epiglottis acted as an umbrella, guiding the water into the esophagus through the clearance between the epiglottis tip and the dorsal pharynx (yellow arrow, fourth panel, Fig. 5). Water was found in the connecting interface between the oropharynx and pharynx (white hollow arrow, second panel, Fig. 5), indicating the high sensitivity of thin liquids like water to geometrical details (including both the anatomical complexities and experimental imperfections).

#### Fast dispense of water to the anterior oropharynx

**Figure 6:**
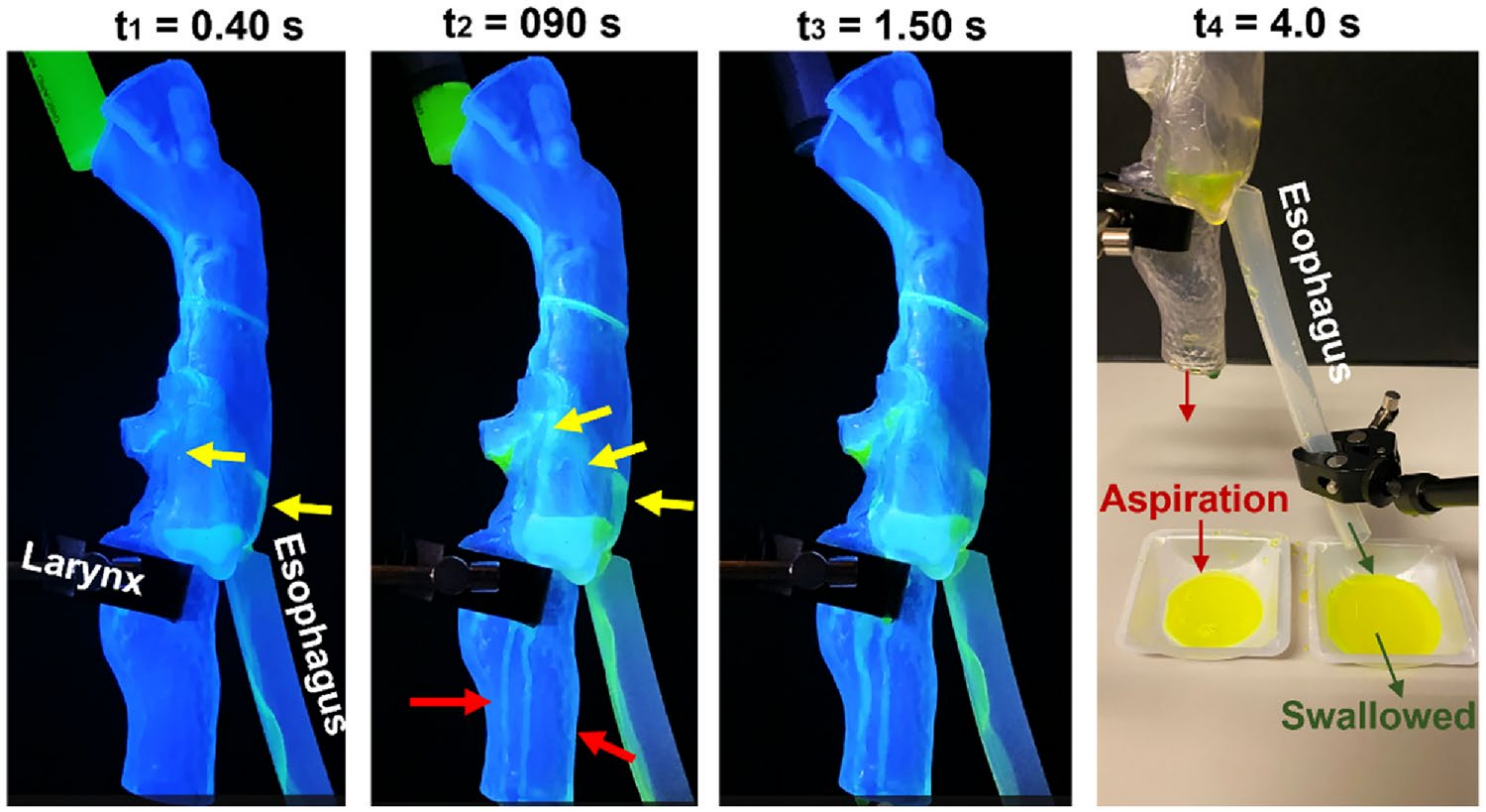
Temporospatial hydrodynamics of water being fast dispensed to the anterior oropharynx. Also shown in the supplemental video recording S1.

**Figure 7:**
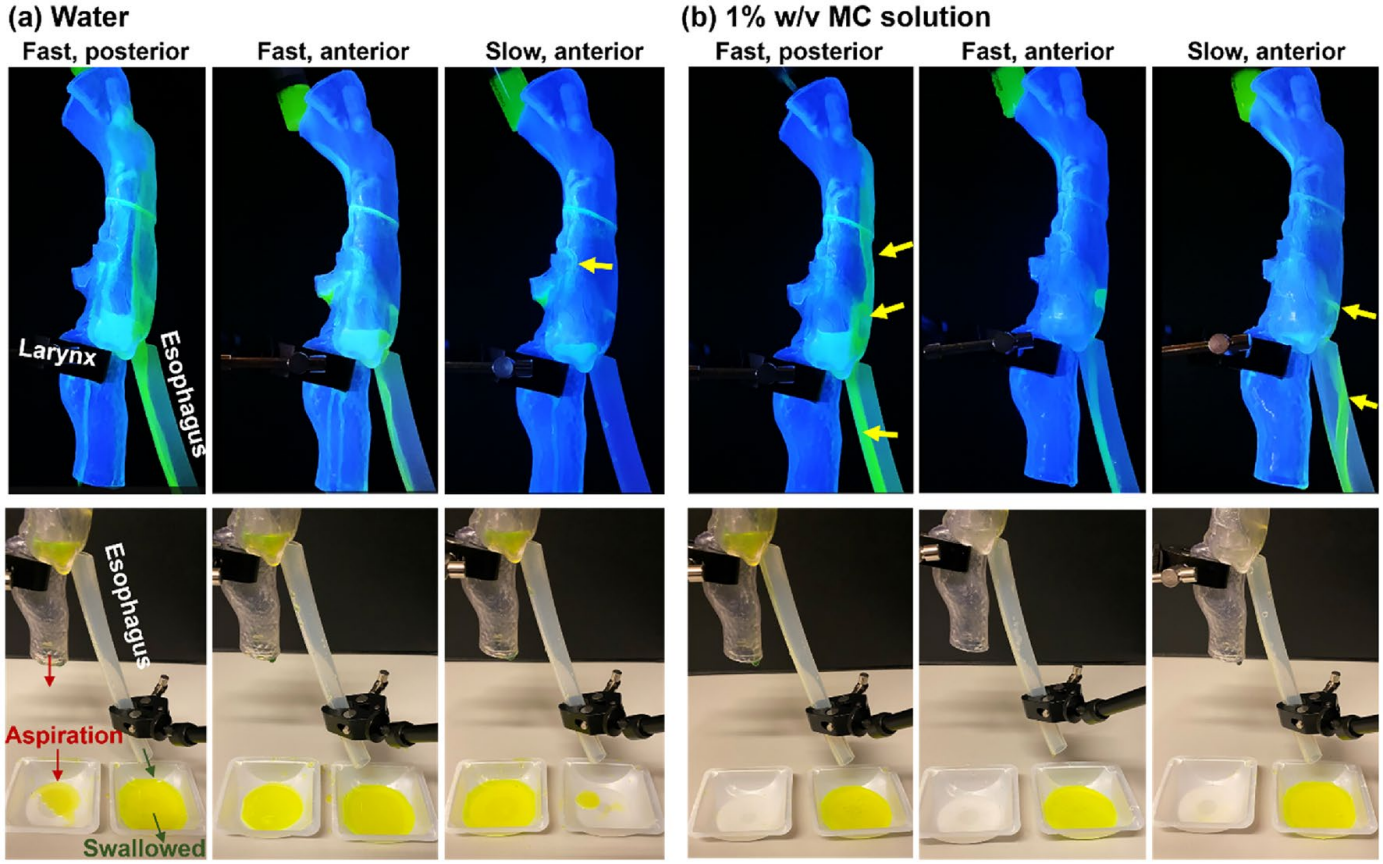
Comparison of liquid hydrodynamics and aspiration under varying dispensing conditions (i.e., fast, posterior; fast, anterior; slow, anterior) for: (**a**) water and (**b**) 1% w/v MC solution. Fast, anterior: fast dispensing to the anterior oropharynx. The hydrodynamics of water with slow-dispensing and the MC solution with fast-dispensing can also be viewed in the supplemental video recording S3 and S4, respectively.

Figure 6, as well as the supplemental video S2, shows the hydrodynamics of fast water dispensing (i.e., 5 mL over 1 s) to the anterior oropharynx in the full-throat model, with the three panels exhibiting the start, middle, and end of water dispensing (indicated by the remaining volume in the syringe). In this case, the effects of gravity and inertia were more pronounced than in the previous case. The water flowed quickly along the tongue base and ventral pharynx. Upon impinging on the upper epiglottis, the water spread over it, with some flowing over the epiglottis edge (or aryepiglottic fold), as demonstrated by the streams on the ventral, lateral, and dorsal walls of the pharynx (yellow arrows, Fig. 6). In particular, the liquid stream along the dorsal wall resulted from the water flowing down the 45°-down-tilt epiglottis and through the clearance between the epiglottis tip and dorsal pharynx. Such a liquid stream was absent when the dispensing speed was slow, which will be discussed in the following section (Fig. 7a, first vs. second panels).

Three aspiration liquid streams were observed in the trachea, with two in the middle and one at the back (red arrows, Fig. 6). A significant amount of aspiration was collected, close to that of deglutition (rightmost panel, Fig. 6). It is noted that the esophageal opening can be larger than that hereof, as the larynx will move upward and forward, enlarging the esophageal opening and increasing the deglutition rate, thus slowing down the liquid buildup in the pyriform sinus and lowering the chance of overflow into the inter-arytenoid notch.

#### Slow dispense of water to the anterior oropharynx

Significant aspirations were observed when dispensing water slowly (i.e., 5 mL within 3 s) to the anterior oropharynx (i.e., slow anterior, as shown in the supplemental video S3). Figure 7a compares the aspired fluid, as well as the hydrodynamics around 1.27 s since the start of dispensing, between the slow-anterior condition (middle panel) and the conditions of fast-anterior (first panel) and fast-posterior (third panel). Due to slow dispensing, surface tension played a more significant role than gravity or kinetic energy. The low speed allowed the liquid stream to cling to the arch of the tongue base, which then diverted to two sides (yellow arrow) when reaching the vallecula and subsequently moved down through the upper pyriform fossa. A substantial fraction of water flowed along the aryepiglottic folds, with some of it entering the laryngeal vestibule via the recesses above and below the cuneiform tubercle. This was evidenced by the larger amount of water collected under the trachea than in the esophagus (second panel, Fig. 7a). Aspiration occurred at the middle lateral trachea wall. The water level in the pyriform sinus remained low, and no overflow via the interarytenoid notch was observed.

### Deglutition tests using 50A SLA full-throat model and 1% w/v MC solution

#### Fast dispense of 1% w/v MC solution to the posterior oropharynx

The first panel in Fig. 7b and the supplemental video S4 show the hydrodynamics of 1% w/v MC solution after fast dispensing to the posterior oropharynx. In contrast to water, no aspiration was observed with the MC solution, either in the trachea or the collecting tray (Fig. 7b vs. a, first panels); this observation was consistent with a much-reduced aspiration risk with a thicker liquid^36,37^. The flow pattern of the MC solution was more continuous and coherent than that of water, as displayed along the dorsal pharyngeal wall and inside the esophagus (Fig. 7b vs. a, first panels). No MC solution was deflected from the posterior oropharynx to the tongue base during dispensing. Thus, there is no lateral penetration into the laryngeal vestibule via the recesses above or below the cuneiform tubercle. The MC solution level appeared to be higher than the interarytenoid notch; however, notch overflow aspiration was not observed, presumably due to the liquid bridging effects caused by its much higher viscosity and slightly higher surface tension than water.

#### Fast dispense of 1% w/v MC solution to the anterior oropharynx

No aspiration was observed with a fast dispensing of 1% w/v MC solution to the anterior oropharynx (second panel, Fig. 7b and supplemental video S4). The MC solution followed a clear trajectory, which first moved along the arch of the tongue base, then the upper surface of the slanted epiglottis, followed by a smooth passage through the clearance between the epiglottis tip and dorsal pharynx before entering the esophagus. The fluid stream appeared to be stable throughout the dispensing phase. In comparison to water with significant leaking over the edges of the epiglottis, no edge leaking was noted for the 1% w/v MC solution.

The draining rate into the esophagus (i.e., deglutition rate) was fast, and the liquid level in the pyriform sinus did not rise over the interarytenoid notch. This might result from the smooth flow stream into the esophagus without forming a film blocking the esophagus opening. One counter-example of this hypothesis was the observation of occasional bulging in the water stream, which was completely absent in the MC fluid stream. Such bulging was caused by the sporadic formation of a water film covering the esophagus opening, which temporarily blocked the flow into it, followed by a sudden film rupture and a surge in deglutition flow rate.

#### Slow dispense of 1% w/v MC solution to the posterior oropharynx

Slow dispensing of MC solution to the anterior oropharynx is shown in the third panel of Fig. 7b. Again, no or negligible aspiration was observed in all three repetitions. The slow dispensing speed allowed more MC solutions to flow along the 45°-down-flapped epiglottis, rather than splitting upon hitting the vallecula and diverting laterally to the upper pyriform fossae. As a result, more fluid moved through the clearance between the epiglottis tip and dorsal pharynx and directly entered the esophagus, minimizing the aspiration risk via notch overflows. Due to a smooth flow into the esophagus, the MC solution level in the lower pyriform fossae was persistently low.

### Visualization of overflows and residuals from other views

To verify the phenotypic aspiration mechanisms proposed in this study, water kinematics around the point of interest were recorded from varying angles, including views from the rear, front, and inside (endoscope). Mechanisms to verify consisted of (1) overflow through interarytenoid notch leading to an aspiration stream along the dorsal trachea, (2) overflow through the cuneiform tubercle recesses leading to an aspiration stream on the lateral trachea, and (3) off-edge capillary flow under the epiglottis leading to an aspiration stream along the ventral trachea. An equivalent verification to the last one was whether there was residual accumulation along the conjunction between the down-folded epiglottis and the laryngeal vestibule edge, which was predisposed to drip into the laryngeal vestibule driven by gravity.

#### Rear view

**Figure 8:**
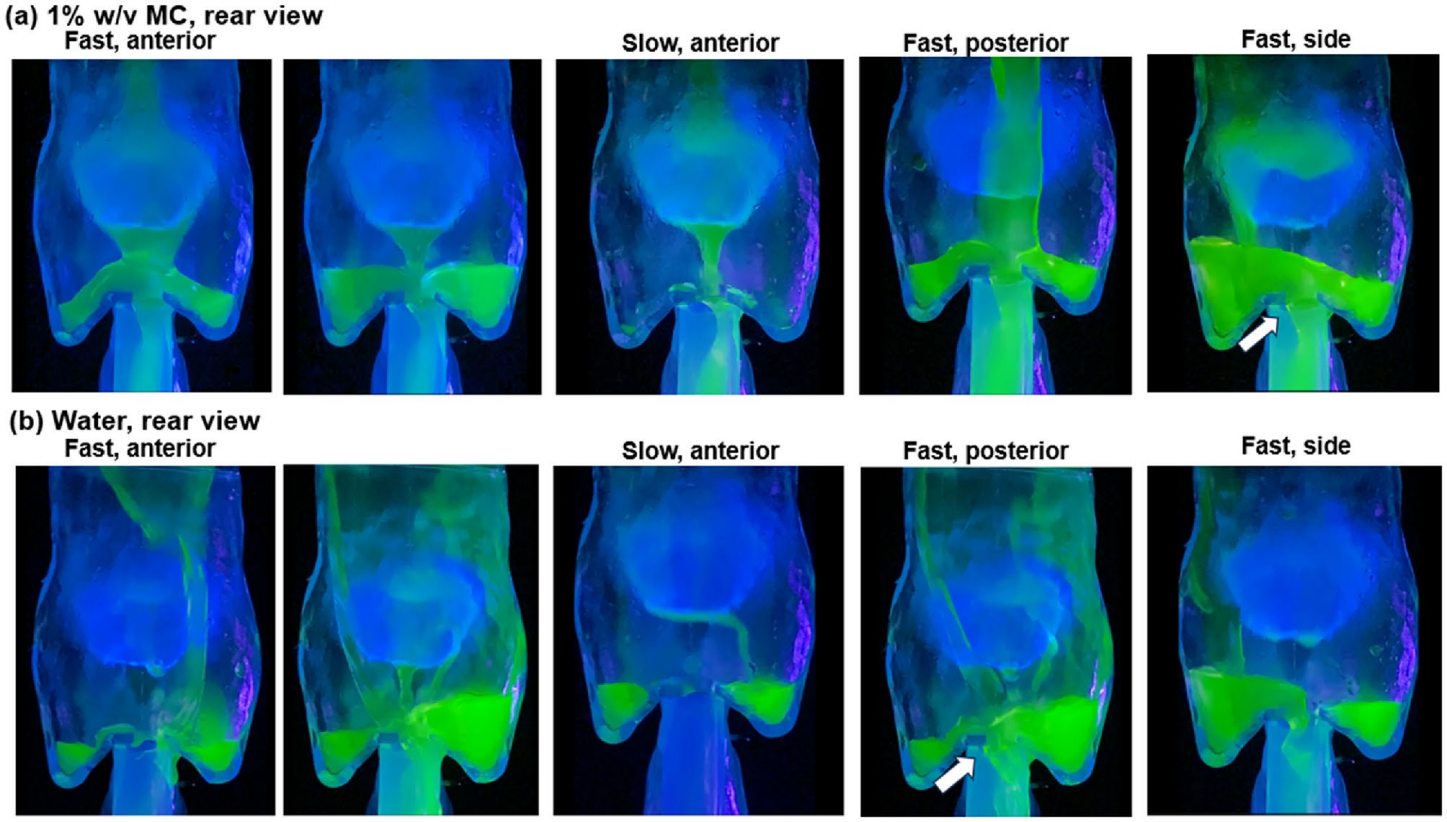
Rear view of the hydrodynamics in the full-throat model with a 45^o^ down-tilt epiglottis: (**a**) 1% w/v MC solution, and (**b**) water. The white arrows denote the occasional formation of a liquid bridge over the esophagus opening. The water motions can also be viewed in the supplemental video recording S5.

Figure 8a,b present the rear view of the hydrodynamics under varying intake conditions for 1% w/v MC solution and water, respectively. For a moderately thick fluid like 1% w/v MC solution, symmetric flow patterns were observed throughout the process, as illustrated by equal liquid levels in the two pyriform sinuses in the first two panels in Fig. 8a. Regular flow patterns were also found for the 1% w/v MC solution under slow dispensing to the anterior oropharynx (third panel) or fast dispensing to the posterior oropharynx (fourth panel, Fig. 8a). One difference between these two cases was the liquid level in the pyriform sinuses, which did not build up under the slow dispensing condition due to esophagus drainage. When dispensed from the posterior oropharynx, the MC solution adhered to the dorsal pharynx and directly entered the esophagus, with a lower likelihood of overflow over the interarytenoid notch and nearly no likelihood for overflow over the cuneiform recesses nor off-edge capillary flows. When dispensed to the side oropharynx, the MC solution mainly flowed through the groove between the lateral pharynx and epiglottis, with a small fraction flowing over the upside epiglottis. The slanted liquid level in the pyriform sinus in the rightmost panel in Fig. 8a indicated a lower esophagus drainage rate than the dispensing rate.

In contrast to the coherent flow patterns for the MC solution, water exhibited more irregular patterns and more complex dynamics. Fast dispensing of water to the anterior oropharynx led to a highly transient process, including stream ramifications, coalescence, and splashing, as shown in Fig. 8b and the supplemental video S5. There were three rivulets at the epiglottis tip in the second panel in Fig. 8b, indicating the significance of the epiglottis edge on the water film dynamics. Note that the off-edge flows could lead to aspirations. The hydrodynamics along the epiglottis edge was crucial in determining whether off-edge aspiration occurred or not and in estimating the amount of aspiration under different conditions (e.g., dispensing rate). Water flowed along the rounded edges of the epiglottis more often than the MC solution for any corresponding conditions (Fig. 8b *vs*. a). Besides, all water flows were observed to be asymmetric (Fig. 8b), as opposed to the symmetry of MC flows (Fig. 8a), indicating unstable hydrodynamics and higher chances for cuneiform recess overflows.

#### Front view of slow dispensing of water to the anterior oropharynx

**Figure 9:**
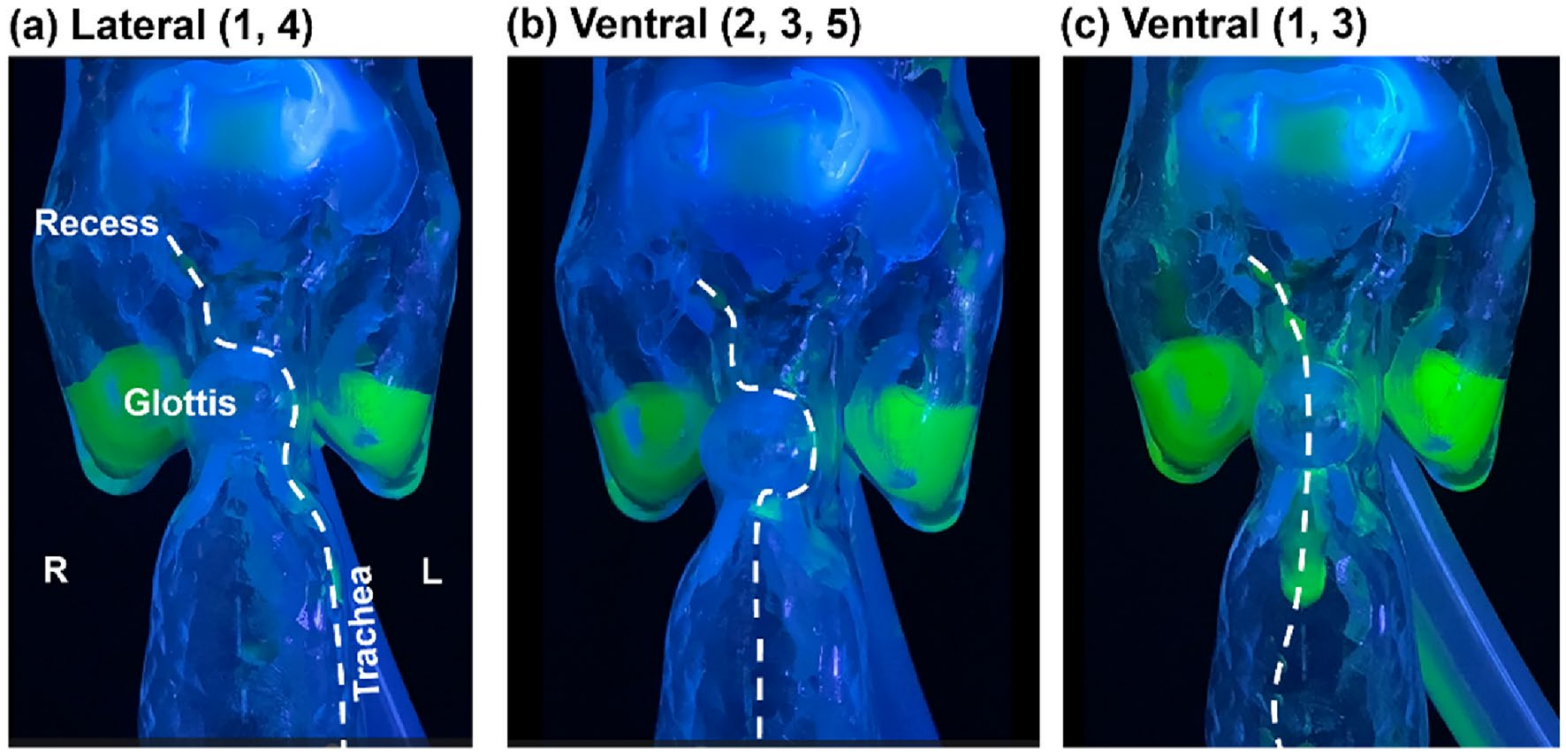
Front view of water hydrodynamics of five test cases (1–5) under slow dispensing to the anterior oropharynx with the aspirating streams observed in: (**a**) lateral trachea (cases 1 and 4), (**b**) ventral trachea (cases 2, 3, 5), and (**c**) ventral trachea with a different drop trajectory (cases 1, 3). The hydrodynamics in case 3 can be viewed in the supplemental video recording S6.

Considering that the lateral and rear views cannot clearly visualize the dynamic fluid interactions with the front throat, we further examined the hydrodynamics with a front view of the worst scenario, i.e., slow dispensing of water to the anterior oropharynx (Fig. 9). Five test runs were undertaken with identical dispensing conditions, and aspirating streams in the trachea were observed in all five cases, confirming the susceptibility of this intake scenario. However, different aspiration symptoms were recorded, with a stream along the lateral trachea in two cases (Fig. 9a) *vs*. along the ventral trachea in four cases, as illustrated by the dashed lines in Fig. 9. Among the five cases with an identical anatomy and intake condition, similarities existed among aspiring liquid streams, i.e., starting from the right cuneiform recess, traveling to the left glottal aperture due to inertia, and bifurcating either to the lateral or ventral trachea (first and second panels, Fig. 9). Further reviewing the video recordings (supplemental video S6) near the glottis also revealed subtle differences among the sources for the aspirating streams along the ventral trachea: the stream starting from the right cuneiform recess traveled directly to the glottis tip and down to the ventral trachea without detouring to the left as in the second panel, likely due to a slower speed of the fluid bolus.

#### Endoscope view of slow dispensing of water to the anterior oropharynx

**Figure 10:**
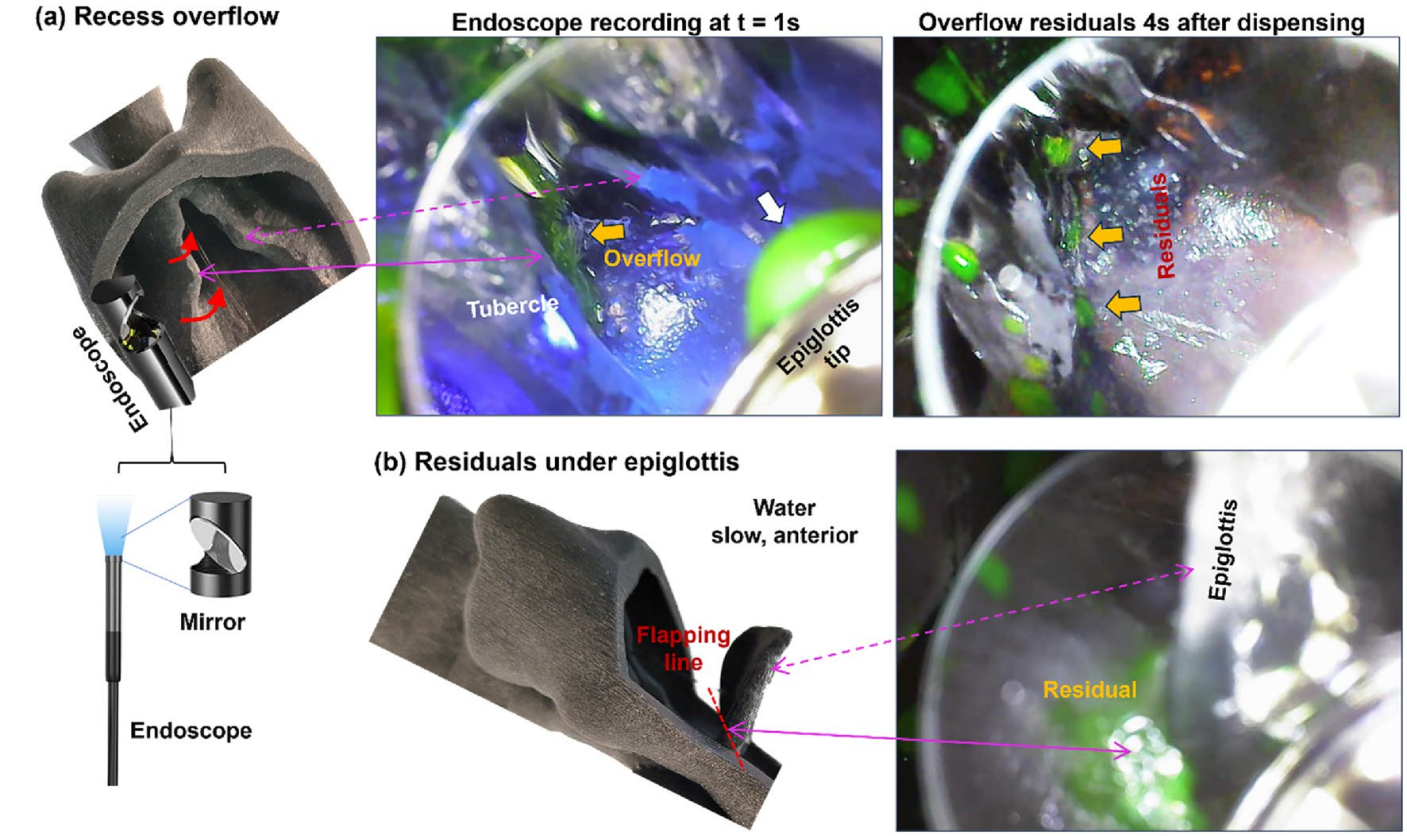
Endoscope imaging for the case with slow dispensing of water to the anterior oropharynx to capture (**a**) overflow through the recesses above and below the cuneiform tubercle and (**b**) residuals under the epiglottis along the flapping line.

Endoscope video recording further verified the overflow of water through the recesses above and below the cuneiform tubercle under the anterior slow dispensing condition, as shown by the brown arrow in the middle panel of Fig. 10a. A static image taken at 4 s after dispensing also exhibited residual drops along the overflow path (three brown arrows in the right panel of Fig. 10a). To help understand the endoscope image, a throat cast was displayed in the same direction as the image, with the endoscope camera in position with a side-view mirror to focus on the laryngeal vestibule (left panel, Fig. 10a). Recess overflows below and above the tubercle were illustrated using two red solid arrows, respectively. A water stream falling off the epiglottis tip was also captured (white arrow in the middle panel of Fig. 10a).

Endoscope imaging also captured water residuals under the epiglottis along the flapping line, as shown in Fig. 10b. These residuals indicated that (1) flow splitting occurred after hitting the vallecula, (2) water moved down the convex epiglottis and flowed over the epiglottis edge, and (3) capillary flow formed underneath the epiglottis along the flapping line due to the acute angle of this space, as well as liquid-wall adhesion and liquid–air surface tension.

### Silicone-molded half-throat model

**Figure 11:**
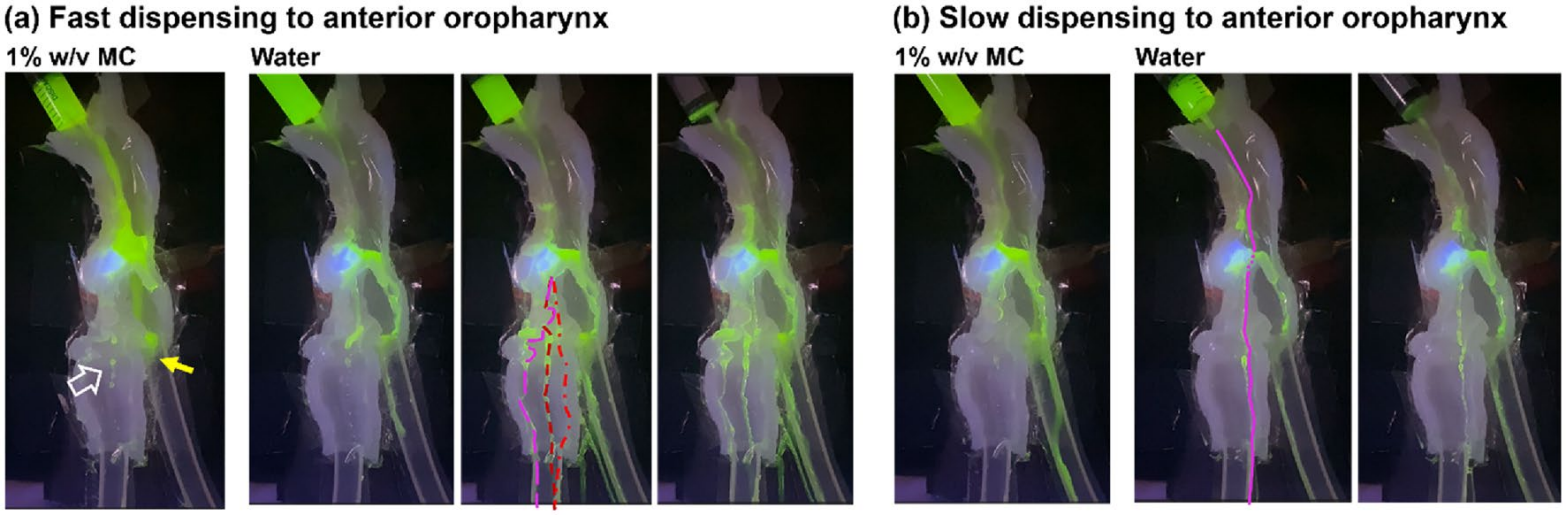
Liquid dynamics in the silicone-molded half-throat model with a 45° down-tilt epiglottis for 1% w/v MC solution and water: (**a**) fast dispensing to the anterior oropharynx, and (**b**) slow dispensing to the anterior oropharynx.

Figure 11 shows the hydrodynamics of dispensed liquids to the anterior oropharynx of the silicone-molded half-throat model. A clearer view of the fluid interactions with the epiglottis was obtained, especially around the vallecula, which was visually obscured when using the full-throat model. When fast dispensed to the anterior oropharynx, the 1% w/v MC solution followed a smooth trajectory along the curvature of the tongue base, diverted into the lateral groove upon reaching the vallecula, and accumulated in the pyriform fossa. Esophagus drainage started when 70% of the MC solution was dispensed (yellow arrow, first panel, Fig. 11a). No splashing was observed throughout the process, and the liquid motion was regular. A careful examination also revealed that the four drops near the pharynx and upper trachea resulted from the clearance between the half model and plexiglass and, thus were not indicative of aspiration or penetration. It should also be noted that slight disparities inevitably existed between the half-throat and full-throat experimental setups; one such difference was that the epiglottis in the half model pressing against the dorsal pharynx while a small clearance existed in the full-throat model.

In comparison to the MC solution, drastically different hydrodynamics were observed when fast-dispensing water to the anterior oropharynx. The water moved much faster from the oropharynx to the esophagus opening, leaving a much thinner coating on the tongue base and vallecula and leading to an earlier onset of esophagus draining (around 20% dispensing). The water motion was more irregular than the 1% w/v solution, and apparent aspirations occurred. Three water streams were observed in the front, middle, and back of the trachea, respectively, as shown by the dashed lines in Fig. 11a. This was consistent with the observations in the compete-throat model (Fig. 7a). The aspirations at the front, middle, and back of the trachea were caused by the epiglottis edge leak (off-edge flow), cuneiform tubercle recess overflow, and interarytenoid notch overflow, respectively. A further examination of the water motion recording confirmed the overflow via the cuneiform tubercle recesses that cause the lateral middle stream in the trachea.

Figure 11b illustrates the hydrodynamics of slow-dispensed liquids to the anterior oropharynx. In this case, no aspiration was observed for the 1% w/v MC solution. However, a water stream was observed in the dorsal trachea, which was verified to be the result of overflow via the cuneiform tubercle recesses, as illustrated by the pink line in Fig. 11b.

### Aspiration quantification

**Figure 12:**
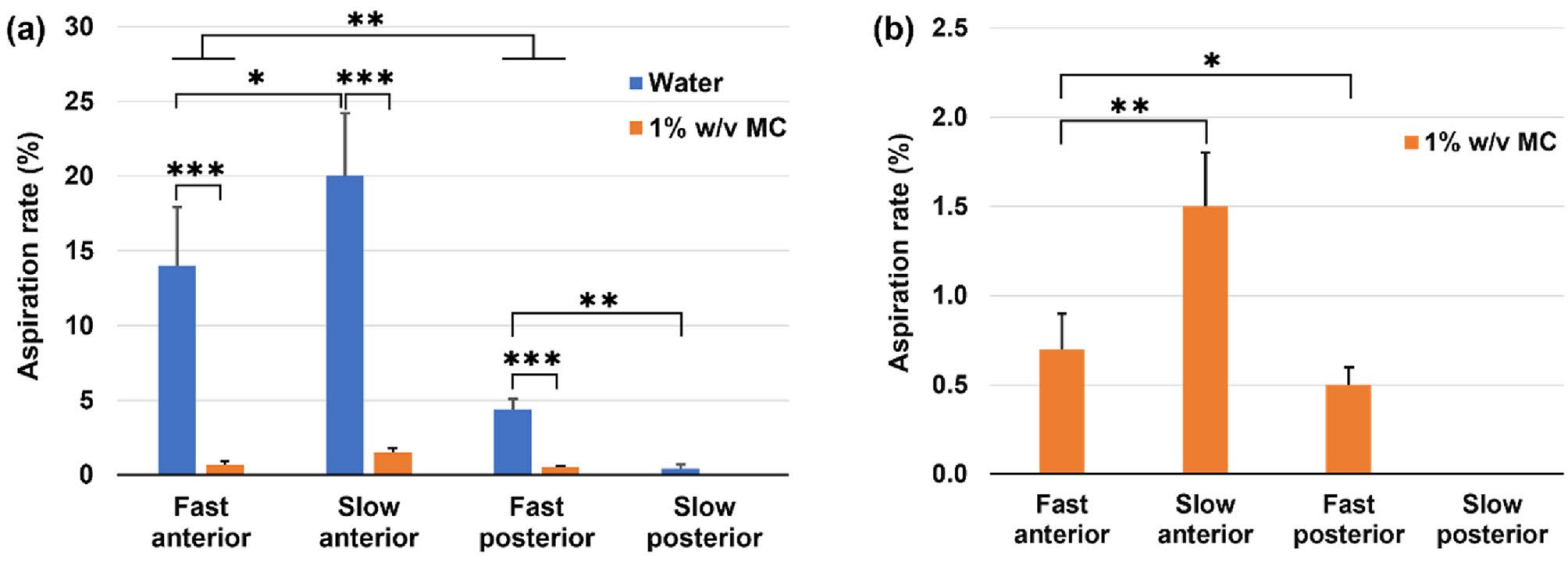
Aspiration quantification for water and 1% w/v MC solution under varying dispensing conditions using the full-throat model.

Figure 12 shows the aspiration rate for water and 1% w/v MC solution under four dispensing conditions. Using the full-throat model in the study, it is observed that water, which is a thin liquid (Level 0), has one order of magnitude higher risk (9–20) for aspiration than the 1% w/v MC solution, which is a mildly thick liquid (Level 2). Among the four dispensing conditions, slow dispensing to the anterior oropharynx gives the highest aspiration risk, while slow dispensing to the posterior oropharynx gives the lowest risk, irrespective of the liquid consistency.

Considering the dispensing site (i.e., tongue retraction) effects, dispensing liquids to the anterior oropharynx has significantly higher aspiration risks. The dispensing speed also has a significant impact on aspiration risks, regardless of the dispensing site or liquid type. However, different trends of the dispensing speed effects are observed depending on the dispensing site. To the anterior oropharynx, slow dispensing increases the aspiration, while to the posterior oropharynx, slow dispensing drastically decreases the aspiration, regardless of the liquid type (Fig. 11a,b).

## Discussion

### Three aspiration mechanisms

In this study, we systematically studied influencing factors in two (full and half) throat-epiglottis models, including liquid consistency (thin and mildly thick), dispensing site (anterior and posterior oropharynx), and dispensing speeds (fast and slow). By examining the liquid trajectories in aspiration cases from recordings of different views (lateral, rear, front, inside), we observed three mechanisms underlying high aspiration risks at the instant of deglutition (i.e., with a 45° down flapped epiglottis), as follows.

1. Liquid overflows into the laryngeal vestibule through the interarytenoid notch. There are two competing factors in the liquid level in the pyriform fossa; one is the liquid descending from the oropharynx increasing the liquid level, and the other is the liquid entering the esophagus decreasing the liquid level. When the liquid line rises over the interaryteniod notch, penetration into the laryngeal vestibule occurs, which further leads to aspiration liquid streams along the dorsal trachea. We termed this phenotype as ‘*notch overflow aspiration’*. It is noted that a liquid line slightly higher than the interaryteniod notch may or may not elicit the notch overflow depending on the fluid’s type. Entry capillary pressure exists that necessitates a larger gradient to initiate overflow and varies with the liquid viscosity, surface tension (liquid–air), and wall adhesion (liquid-wall). In this study, notch overflow of water is observed to be much easier than the 1% w/v MC solution, the latter of which has both higher viscosity and surface tension. It is also noted that, due to the same reason, a liquid film bridge can occasionally form over the esophagus opening (denoted by white arrows in Fig. 8a,b), which interrupts the liquid drainage and drives the rise in liquid level in the pyriform fossa, thus increasing aspiration risk.
2. Liquid overflows into the laryngeal vestibule via the recesses above or below the cuneiform tubercle. When the fluid descends following the upper pyriform fossa (i.e., the passage between the epiglottis and lateral pharynx), chances exist for the liquid flowing along the aryepiglottic fold to overflow into the laryngeal vestibule through the two recesses (or indentations) located above and below the cuneiform tubercle, as shown by the endoscope imaging in Fig. 10. This chance will become higher for thinner liquid with lower surface tension. We termed this phenotype as ‘*recess overflow aspiration’*. In this study, the recess aspiration rate for water was observed to be one order of magnitude higher than that for the 1% w/v MC solution (Fig. 12a). The ensuing aspiration stream can often be seen in the middle lateral trachea (Figs. 7, 11).
3. Some fluids fall over the side edges of the convex epiglottis, cling to the underside of the epiglottis via capillary force, and eventually move into the laryngeal vestibule driven by gravity. This aspiration stream, termed '*off-edge aspiration’*, can be frequently seen in the ventral trachea and, less often, in the dorsal trachea (Fig. 9).

Besides the three types of aspirations, significant residuals were also found under the epiglottis along the flapping line using an endoscope (Fig. 10b). This residual would likely enter the laryngeal vestibule driven by gravity, causing aspiration.

### Relative significance of influencing factors

All factors considered in this study (liquid type, dispensing site, and speed) showed a noticeable impact on penetration/aspiration risks. Among them, liquid viscosity (1 mPaˑs for water, thin *vs*. 245 mPaˑs for 1% w/v MC solution, mildly thick) was observed to exert the most significant impact on both flow dynamics and aspiration rate. In comparison to the highly complex flow patterns of water passing the throat-epiglottis passage, the MC solution appeared to be smoother, slower, and more coherent without the splashing and wavy flows observed in water flows (Figs. 7, 8, 11). The aspiration rate from water was quantified to be one order of magnitude higher than the MC solution for both dispensing sites and speeds considered (Fig. 12). A further multi-view examination of the flows entering the laryngeal vestibule revealed that water was much more likely to elicit aspiration through the three mechanisms, i.e., notch overflow, recess overflow, and off-edge capillary flow (Figs. 9 and 10). Thus, adjusting liquid consistency or food texture will still play an important role in dysphagia management.

In this study, the dispending site (anterior *vs*. posterior oropharynx) exerted a larger impact on aspiration risk than the dispensing speed (fast *vs*. slow, or 5 mL over 1 s vs. 3 s). Varying the dispensing site or speed mainly changed the liquid’s path from the oropharynx to the pyriform fossa, which further changed the relative magnitudes of the three aspiration mechanisms. For instance, when dispensing a liquid to the anterior oropharynx, the majority of it would descend along the tongue base curvature, divert laterally into the upper pyriform fossa upon reaching the vallecula, and accumulate in the pyriform sinuses before entering the esophagus. During this process, all three aspiration phenotypes were observed (Figs. 7a and 11a), with off-edge aspirations from the convex epiglottis, recess overflow aspirations from fluids moving along the aryepiglottic fold within the upper pyriform fossa, as well as the notch overflow when the liquid level in the pyriform sinuses went beyond the interarytenoid notch. On the other hand, the majority of liquid flowed along the dorsal pharynx during posterior dispensing, giving rise to a smoother entry into the esophagus and a lower aspiration rate (Figs. 7 and 12). In this case, the aspirations were from either notch overflows or recess overflows, but not off-edge flows. The flow paths also differed when dispensed at different speeds, as illustrated by the flow pattern differences between 'fast, anterior' and 'slow anterior' for water and 1% w/v MC solution in Fig. 7a,b. However, these differences appeared much less significant than those arising from anterior–posterior dispensing.

**Figure 13:**
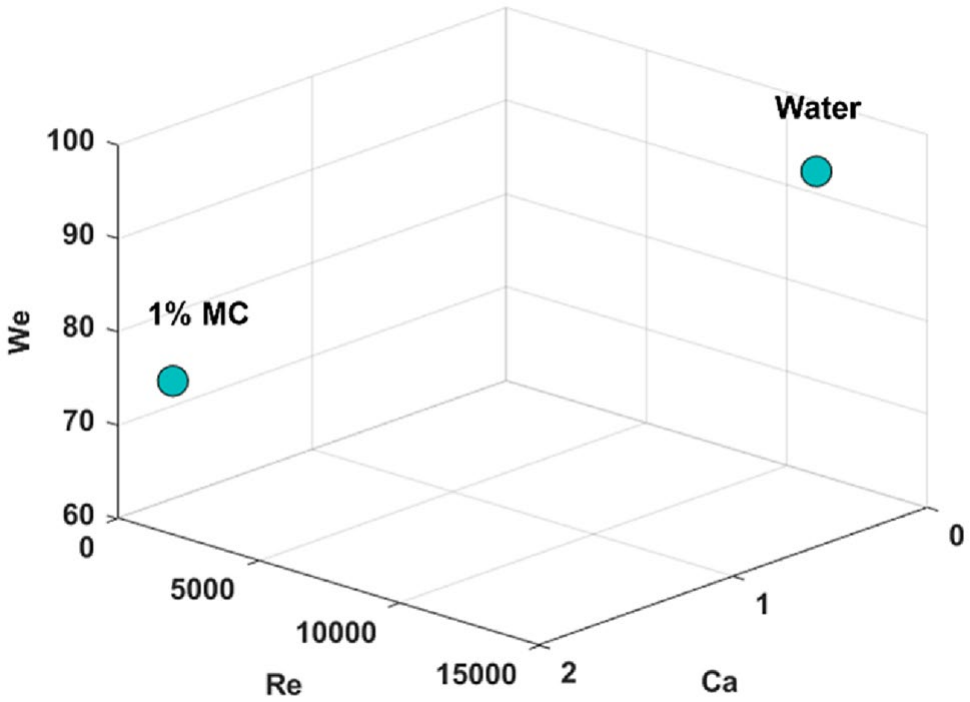
Fluid diagram showing key nondimensional parameters of Re, Ca, and We.

Dimensional analyses of the fluid dynamics in the pharynx for water and 1% MC solution. The width of the epiglottis was selected as the characteristic length, which was measured as 18.5 mm. Considering the complex fluid flow patterns, only the maximal speed of the fluid bolus at the beginning of liquid dispensing was measured, where the front of the fluid bolus was tracked in consequential frames of the video. The maximal bolus speed was found in the pharynx, which was about 0.60 m/s for water and 0.56 m/s for the 1% MC solution. Using the viscosity and surface tension measured in this study, three nondimensional parameters, i.e., Reynolds number (Re = ρVL/µ), Weber number (We = ρV^2^L/σ), and capillary number (Ca = µV/σ) were calculated, as listed in Table 2 as well as in Fig. 13 as a 3D plot. The Weber number is useful in analyzing the behavior of fluids with free surfaces and determining the influence of surface tension forces relative to inertial forces. The We values are large (72.7–92.5), indicating that inertia dominates over surface tension, leading to breakup or deformation of the fluid bolus, which is particularly obvious for water, as observed in Figs. 5 and 6. The Capillary number Ca differs significantly between these two fluids, being 0.0083 for water and 1.72 for the 1% MC solution. Regarding the relative force magnitudes, it is observed that for water, inertia >  > surface tension >  > viscosity, while for the 1% MC solution, inertia >  > surface tension ≈ viscosity. In Fig. 13, these two fluids are located in distinct regions, indicating very different behaviors between them. Clearly, the large viscous effect in the 1% MC solution stabilizes the flow, reducing the breakups of the fluid bolus despite its continuous morphological deformation, as displayed in Fig. 8.

**Table 2:** Dimensional analysis of liquid flows in the pharynx (epiglottis width: L = 18.5 mm).

	ρ (kg/m^3^)	V_max_ (m/s)	µ (Paˑs)	σ (N/m)	Re	We	Ca
Water	1000	0.60	0.001	0.072	11,100	92.5	0.0083
1% MC	1000.1	0.56	0.245	0.079	42.3	72.7	1.72

### Comparison to existing and new methods in visualizing dysphagia

Visualization of anatomy and bolus kinematics is still the major, if not sole, method in diagnosing dysphagia^59^. The two primary approaches are Fiberoptic Endoscopic Evaluation of Swallowing (FEES) and Modified Barium Swallow Study (MBSS) or Videofluoroscopic Swallow Study (VFSS). In MBSS/VFSS, real-time X-ray videos are captured as patients swallow food/liquid mixed with barium to visualize the bolus path and detect aspiration^60^. This is similar to the lateral view of fluid dynamics in the throat-epiglottis models aided with fluorescent dye and LED lighting. In particular, the half-throat model shared even closer similarities with MBSS/VFSS in clearly revealing fluid behaviors and fates in the mid-sagittal plane of the pharyngolaryngeal tract (Fig. 11).

The results of this study demonstrate that lateral visualization using the full-throat model can provide new information on swallowing and aspiration, which may be missed by MBSS/VFSS, a two-dimensional technique through the middle sagittal plane. Such information includes descending flows in the upper pyriform fossa, penetration flows over the cuneiform tubercle recesses, fluid accumulations in the lateral pyriform sinus, and aspiration flow streams on the lateral trachea wall, as illustrated in Figs. 5, 6 and 7. Additionally, the rear and front views depicted in Figs. 8 and 9 offered further insights into the hydrodynamics passing the epiglottis and glottis, respectively, allowing for a comparison of the hydrodynamics between the two lateral sides.

The endoscopic view in Fig. 10 provides the needed verification for the recess overflow mechanism, clearly showing that descending fluids in the upper pyriform fossa enter the laryngeal vestibule through the recesses above or below the cuneiform tubercle. This recess overflow mechanism can be inferred from the aspiration streams on the lateral trachea, but is not observable from either the lateral, rear, or front views because the recesses are enclosed by the pharyngeal walls. The setback of this approach, similar to in vivo FEES, is image distortion, invasive disruption to flow and structural motions, and difficulty in deployment^61,62^.

It is worth noting that new methods have been introduced in recent years to study dysphagia, namely computational simulations and machine learning. Michiwaki et al. (Michiwaki et al., 2020) developed a computational model containing both the airway and tissues based on CT images and studied the bolus flow dynamics. They observed that fluid dispensed to the posterior oropharynx has higher aspiration risks than the anterior-dispended fluid, which appears contradictive to what we observed in this study. However, a closer comparison revealed that this seeming contradiction was attributed to a delayed epiglottis down-flapping in their model. The uptilt epiglottis, at the instant of dispensing, diverted the posterior-dispended fluids to the anterior pharynx and vice versa^63^. This led to similar flow paths between their posterior(anterior)-dispensed fluids and the anterior(posterior)-dispensed fluids in this study, which featured an epiglottis tilted downward at 45°. On the other hand, machine learning, and deep learning in particular, have unfolded their strengths in the automatic analysis of VFSS/FEES images and classifying dysphagia phenotypes^64–66^. Non-invasive assessment of swallowing based on externally measurable signals provides another exciting alternative. Yagi et al.^67^ developed a dysphagia monitoring system utilizing respiratory flow, swallowing sound, and laryngeal motion, and found that the laryngeal activation duration correlated well with the Videofluoroscopy healthy participants but not in dysphagia patients.

## Limitations

Following a bottom-up approach, this study made several simplifications, including a static throat, no oral cavity, and models from one patient. Normal oropharyngeal swallowing comprises a sequence of precisely coordinated motions of the tongue, thyroid, dorsal pharynx, larynx, and epiglottis. At least 26 pairs of bilateral muscles in the upper aerodigestive tract and five cranial nerves are involved in swallowing motor and sensory functions^68^. Attempting to include all or most of these factors is not only formidable (if not infeasible at all), but also poses challenges in identifying individual anatomy-function causal relations underlying phenotypic swallowing disorders. Adopting a highly accurate, static throat anatomy with a down-flapped epiglottis is similar to a "freeze" condition recommended by Fanucci et al.^34^ that enables the evaluation of swallowing details. It is also acknowledged that excluding the structure motions can only partially mimic the life condition and needs to be considered later on top of the current study. We envision that adding more factors will mainly change the relative magnitudes of the three aspiration mechanisms but are not likely to add new mechanisms. For instance, including a weaker relaxation of the cricopharyngeal muscle will reduce the esophagus drain rate, resulting in quicker liquid accumulation in the pyriform fossa and higher risks for notch overflow aspiration. Other dynamic structures not considered hereof include tongue-pharynx constriction, epiglottis flapping, hyoid forward motion, and upward-forward motion of the larynx^69,70^. Of note, both ends of the model were open to the atmosphere and thus no significant internal pressure buildup in the pharynx was expected. However, a precise coordination of pressures within the pharynx is essential for safe swallowing and abnormalities in pressure dynamics can lead to swallowing disorders and increase the risk of aspiration, necessitating the inclusion of both ends and coordinated structural motions^71^. The bolus transport in the oral cavity is one of the three swallowing phases per se, determining how the bolus enters the oropharyngeal tract^72,73^. In this study, this process was considered by means of two dispensing speeds and sites to the oropharynx. Dedicated investigations into oral swallowing can be viewed in two previous studies, where the impacts of liquid’s rheological properties were systemically evaluated on bolus dynamics^74,75^. Intersubject variability in anatomy exists between health and disease and among ages and genders; thus, larger cohorts of throat-epiglottis models are warranted in future studies.

## Summary

With transparent, anatomically accurate throat-epiglottis models, fluid motions under various swallowing scenarios were visually examined, and ensuing aspiration were quantified. Three aspiration mechanisms were identified with corresponding representations in the trachea. Specific findings are listed below.

1. Fluid bolus entered the laryngeal vestibule by three mechanisms: (a) overflow via the interarytenoid notch (notch overflow), (b) overflow via the recesses above/below the cuneiform tubercle (recess overflow), and (c) off-edge flow under the epiglottis dripping into the laryngeal vestibule (off-edge capillary flow).
2. Water had much higher aspiration risks than 1% w/v MC solution (mildly thick) regardless of the liquid dispensing site and speed due to stronger notch and recess overflows.
3. When a liquid bolus flows at a very slow rate along the anterior pharyngeal wall, there is a high risk of aspiration due to off-edge capillary flows.
4. The impact of dispensing speed varied. A slower speed increased aspiration for liquids dispensed anteriorly due to increased off-edge capillary flows, while it significantly decreased aspiration for posteriorly dispensed liquids, attributable to a smoother entry into the esophagus and minimized overflow through the interarytenoid notch.
5. Of the three factors evaluated, liquid viscosity had the most pronounced effect on the aspiration rate. This is followed by the dispensing location and then the dispensing speed.

### Supplementary Information

Supplementary Video 1.

Supplementary Video 2.

Supplementary Video 3.

Supplementary Video 4.

Supplementary Video 5.

Supplementary Video 6.

Supplementary Information 1.

## Data Availability

The data that support the findings of this study are available from the corresponding author upon reasonable request.
