# Supplementary material for: Oropharyngeal swallowing hydrodynamics of thin and mildly thick liquids in an anatomically accurate throat-epiglottis model
Source: Sci Rep. 2024 May 25;14:11945. doi: 10.1038/s41598-024-60422-x (PMC11126673; doi:10.1038/s41598-024-60422-x)
Supplement: Supplementary file 7 — Supplementary Information 1. [file 41598_2024_60422_MOESM7_ESM.docx]

**Supplementary File**

**Videos of oropharyngeal swallowing hydrodynamics of thin and mildly thick liquids**

1. The video S1 (S1_Lateral_Water_Fast_Posterior_Fig5.mp4), shows the lateral view of the water motion that was dispensed quickly (i.e., 5 ml within 1 s) to the posterior oropharynx. This video is supplementary to Fig. 5.
2. The video S2 (S2_Lateral_Water_Fast_Anteriort_Fig6.mp4), shows the lateral view of the water motion that was dispensed quickly (i.e., 5 ml within 1 s) to the anterior oropharynx. This video is supplementary to Fig. 6.
3. The video S3 (S3_Lateral_Water_Slow_Anterior_Fig7a.mp4), shows the lateral view of the water motion that was dispensed slowly (i.e., 5 ml within 3 s) to the anterior oropharynx. This video is supplementary to Fig. 7a.
4. The video S4 (S4_Lateral_MC_Fast_Posteriort_ig7b.mp4), shows the lateral view of the 1%-MC solution motion that was dispensed quickly (i.e., 5 ml within 1 s) to the posterior oropharynx. This video is supplementary to Fig. 7b.
5. The video S5 (S5_Rear_Water_Fast_Anterior_Fig8b.mp4), shows the rear view of the water motion that was dispensed quickly (i.e., 5 ml within 1 s) to the anterior oropharynx. This video is supplementary to Fig. 8b.
6. The video S6 (S6_Front_Water_SLow_Anterior_Fig9b.mp4), shows the front view of the water motion that was dispensed slowly (i.e., 5 ml within 3 s) to the anterior oropharynx. This video is supplementary to Fig. 9b.
